# The Effect of Medium Chain Triglycerides on Time to Nutritional Ketosis and Symptoms of Keto-Induction in Healthy Adults: A Randomised Controlled Clinical Trial

**DOI:** 10.1155/2018/2630565

**Published:** 2018-05-22

**Authors:** Cliff J. d C. Harvey, Grant M. Schofield, Micalla Williden, Joseph A. McQuillan

**Affiliations:** ^1^Human Potential Centre, AUT University, Auckland, New Zealand; ^2^Health, Sport and Human Performance, University of Waikato, Hamilton, New Zealand; ^3^Sports Performance Research Institute New Zealand, AUT University, Auckland, New Zealand

## Abstract

Medium chain triglycerides (MCTs) are ketogenic and might reduce adverse effects of keto-induction and improve time to ketosis and the tolerability of very low carbohydrate diets. This study investigates whether MCT supplementation improves time to nutritional ketosis (NK), mood, and symptoms of keto-induction. We compared changes in beta-hydroxybutyrate (BOHB), blood glucose, symptoms of keto-induction, and mood disturbance, in 28 healthy adults prescribed a ketogenic diet, randomised to receive either 30 ml of MCT, or sunflower oil as a control, three times per day, for 20 days. The primary outcome measured was the achievement of NK (≥0.5 mmol·L^−1^ BOHB). Participants also completed a daily Profile of Mood States and keto-induction symptom questionnaire. MCT resulted in higher BOHB at all time points and faster time to NK, a result that failed to reach significance. Symptoms of keto-induction resulted from both diets, with a greater magnitude in the control group, except for abdominal pain, which occurred with greater frequency and severity in the MCT-supplemented diet. There was a *possibly beneficial* effect on symptoms by MCT, but the effect on mood was *unclear*. Based on these results, MCTs increase BOHB compared with LCT and reduce symptoms of keto-induction. It is unclear whether MCTs significantly improve mood or time to NK. The trial was registered by the Australia New Zealand Clinical Trial Registry ACTRN12616001099415.

## 1. Introduction

Very-low-carbohydrate ketogenic diets (VLCKDs) appear to offer specific benefits for health conditions ranging from neurological disorders, cancer, and obesity, diabetes, and other metabolic conditions [1–11]. A restriction of carbohydrate, either by fasting or by restricting dietary carbohydrate, results in reduced insulin levels, thereby reducing lipogenesis, fat accumulation, and glycogen reserves. Long-chain fatty acids derived from common dietary lipids are almost always bound to albumin and are unable to cross the blood-brain barrier for use as fuel. Thus, when glycogen reserves become insufficient to supply the glucose requirement of the central nervous system (CNS), and for fat oxidation, an alternative fuel is required. During carbohydrate restriction, acetoacetate accumulates and is converted into acetone and beta-hydroxybutyrate (BOHB), leading to the presence of these ketones in the blood and urine (ketonaemia and ketonuria, resp.) and in the breath. Ketone bodies are utilised by tissue as a source of energy, with acetoacetate, the primary ketone body, and BOHB, not technically a ketone body (as the ketone moiety has been reduced to a hydroxyl group), functions as the primary fuel during ketosis resulting in two molecules of acetyl-CoA which enter the Krebs cycle. In ketosis, blood glucose (BG) levels stay within normal physiological limits due to the creation of glucose from glucogenic amino acids and via the liberation of glycerol during fatty acid oxidation.

Nutritional ketosis (NK) results from VLCKDs, as compared to starvation ketosis seen in fasting, and pathological ketosis such as the diabetic ketoacidosis results from uncontrolled Type 1 diabetes [12]. Both starvation or fasting ketosis and nutritional ketosis result from evolutionary adaptations that allowed humans to survive in the absence of carbohydrate foods, and thus, glucose provision [13]. Nutritional ketosis allows for the maintenance of ketosis without starvation, and so NK allows for the maintenance of ketosis for longer than would be achievable with fasted ketosis. VLCKDs typically result in BOHB levels of ≥0.5 mmol·L^−1^ [14], and this level has been used as a cutoff point for entry into NK [15]. Adaptation to NK when transitioning from a standard, higher carbohydrate diet to a VLCKD can cause various short-term adverse effects [16]. These effects of “keto-induction” are constipation, headache, halitosis, muscle cramps, diarrhoea, general weakness, and rash [17]. These occur due to increased natriuresis, kaliuresis, and diuresis in response to lowered insulin levels [18–21] greatest between days 1 and 4 of a fast or ketogenic diet [18]. Transient reductions in glucose provision to the brain have been observed between days 1 and 3 with BG normalising after day four [22], while constipation and other gastrointestinal effects result from reduced food volume, increased fat intake, or reduced fibre intake [23, 24]. Difficulties with adherence to ketogenic diets have been noted [3, 17], but few studies specifically describe early adverse symptoms associated with keto-induction.

Ketogenic diets typically contain a 3 : 1 to 4 : 1 ratio of lipids to nonlipid macronutrients, or at least 75% of calories coming from lipids, very low carbohydrates (often less than 50 g), and low-to-moderate amounts of protein. The 4 : 1 lipid to nonlipid ratio ketogenic diet pioneered at Johns Hopkins University Hospital [25, 26] is now commonly used to induce ketosis and is referred to as a “classic” or “standard” ketogenic diet. Huttenlocher and colleagues first demonstrated that diets containing 60%–75% of calories from lipids induce NK if they include a high proportion of medium chain triglycerides (MCTs) [27]. A VLCKD with 60% of calories derived from MCTs, a three-fold greater intake of carbohydrate (18% versus 6%) and a ∼50% (7% versus 10%) increase in protein, induced NK with little clinical difference in BOHB levels when compared to a standard ketogenic diet [28]. Unlike long-chain triglycerides (LCTs), MCTs do not require the actions of bile nor micellar-chylomicron-mediated absorption into the lymphatics and instead are diffused into the hepatic portal vein and preferentially converted into bio-available ketone bodies in the liver. Moreover, dietary MCTs promote ketonaemia and ketogenesis in both animals [29, 30] and humans [31].

Based on existing evidence, MCT supplementation is demonstrably ketogenic [29–31], increases BOHB in a linear and dose-dependent manner [32–36], and allows the achievement of ketosis with lower amounts of lipids (and concomitantly higher levels of protein and carbohydrate) [28, 37, 38]. However, there is a paucity of research considering the role that MCTs may play in inducing ketosis more rapidly in a ketogenic diet with a 4 : 1 lipid to nonlipid ratio, or in improving symptoms of keto-induction and mood. NK is defined by the magnitude of ketonaemia (specifically ≥0.5 mmol·L^−1^ of BOHB), and symptoms of keto-induction occur during the transition from a standard diet (with limited ketone expression in the blood) and the achievement of NK. Therefore, it is likely that the use of MCTs, resulting in ketonaemia and ketogenesis, could reduce time to NK and symptoms of keto-induction. These symptoms are likely to relate at least in part to the transition from a glucose dominant fuel system to one in which BOHB becomes a primary fuel source. Further, by reducing the time to NK, compliance to a VLCKD could be improved. Likewise, mitigation of symptoms of keto-induction is likely to result in improved adherence to the diet.

The aim of the present study, therefore, was to investigate, in a randomised, double-blind, placebo-controlled trial, whether MCTs reduce time to nutritional ketosis and symptoms of keto-induction and mood in a classic ketogenic diet. The primary outcome measured was the time taken to achieve NK. Secondary outcomes were symptoms and mood.

## 2. Materials and Methods

**Figure 1:**
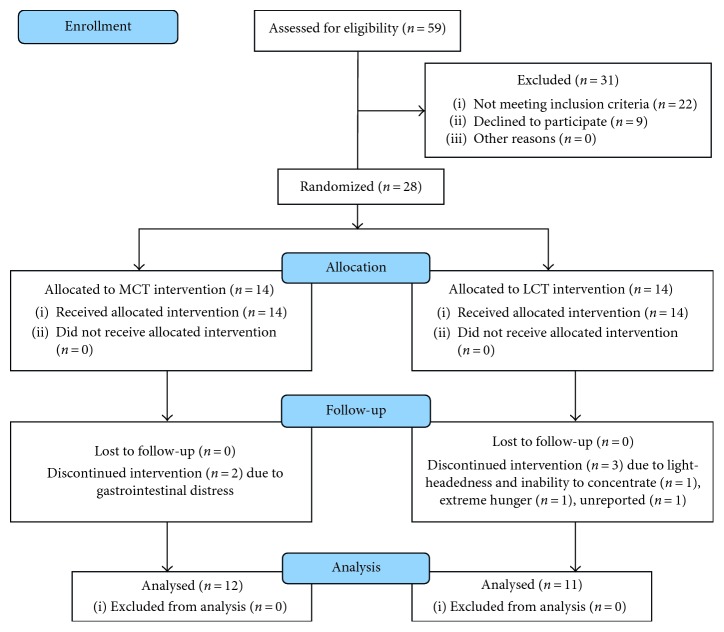
CONSORT flow diagram showing recruitment and retention of study participants.

Twenty-eight participants (2 males, 26 females: age ± SD: 35 ± 4 y) (Table 1) were recruited between the 18th and 19th of October 2015 and gave written, informed consent to participate in this randomised, double-blinded, placebo-controlled study. Participants were required to be nonobese (<30 BMI), not diagnosed as diabetic, not currently nor previously following a ketogenic diet, and not a client of any of the researchers in clinical practice. The study took place between the 2nd and 21st of November 2015. Collection of data and analysis was performed at AUT Human Potential Centre, Auckland, New Zealand (Figure 1).

Participants were prescribed a ketogenic diet with a 4 : 1 lipid to nonlipid ratio. Males were allocated a diet containing 2200 kcal·day^−1^ and females 1800 kcal·day^−1^ each equating to 80% calories from fat (including supplemental oils), 13 to 17% from protein and 3 to 6% from carbohydrate. Minor differences in carbohydrate and protein were due to the use of a protein intake of 1.4 g·kg^−1^ bm·day^−1^ (population means for male and female, resp.), consistent with International Society of Sports Nutrition (ISSN) guidelines for optimal protein intake for performance [39]. Participants were randomised to receive either an MCT supplement containing 65% caprylic acid (C: 8) and 35% capric acid (C: 10) triglycerides (Amtrade NZ limited) or a long chain triglyceride (sunflower) oil (Home Brand), 30 ml, three times per day, for 20 days. While MCTs are essentially nontoxic [40], Ivy et al. observed that 100% of participants in their study experienced gastric distress (cramping and diarrhoea) with dosages of 50 and 60 g MCT, with only small GI effects noted at 30 g [41]. We used this dosage (30 g) as it was most likely to elicit an effect without unduly exposing participants to adverse effects arising from MCT ingestion. For our analysis, we considered the day in which each participant achieved ≥0.5 mmol·L^−1^ BOHB as the time at which they had achieved NK. We proposed this threshold for NK (≥0.5 mmol·L^−1^) based on the level of BOHB observed in those following a VLCKD[14] and as used as a cutoff point for ketosis by Guerci and colleagues [15].

Participants were provided with a “FreeStyle Neo” blood-prick ketometer/glucometer (Abbott Industries) and were required to use the device to measure and record fasted BOHB and BG daily upon waking. Participants were also instructed to complete a questionnaire including a keto-induction symptoms questionnaire (Symptom-Q) and Profile of Mood States-Short Form (POMS-SF). The Profile of Mood States is a questionnaire commonly used to determine the overall mood state of study participants [42]. Saya Shacham developed a shortened, 37 question version of this form with correlation coefficients between the short and original scales all above 95 indicating the suitability of this shortened form for estimating mood. The symptoms questionnaire was developed by one of the authors (d C. Harvey) based on symptoms commonly observed in previous studies of ketogenic diets. The questionnaire asked “In the past 24 hours to what extent have you experienced the following symptoms?” answered on a Likert scale of (1) not at all, (2) mild, (3) moderate, (4) severe, and (5) intolerable for the following symptoms/effects: headache, constipation, diarrhoea, stomach or intestinal pain, intestinal bloating, halitosis (bad breath), muscle cramps, muscle weakness, skin rash, and difficulty concentrating.

An AUT University staff member who was not involved in data collection randomised participants using a simple randomisation technique of coin flipping to generate a treatment sequence into two groups “A” and “B” and labelled the supplemental oils “A” or “B” for distribution and blinding and retained the blinding key. The primary researcher was unblinded to the supplement-oil key only after data analysis had been completed.

Participants were instructed to contact the primary and tertiary researchers for any assistance during the study duration. CH is a registered clinical nutritionist with the New Zealand Clinical Nutrition Association, and MW is a registered nutritionist with the Nutrition Society of New Zealand. The research was conducted in accordance with AUT ethical guidelines. Ethics approval was provided by the AUT University Ethics Committee (approval number 15/317).

### 2.1. Statistical Analyses

Magnitude-based inferences (MBIs) were used to compare the observed measures (BOHB, BG, Symptoms-Q, and POMS-SF). Pairwise comparisons were made between each of the 19 time points for control (LCT) and experimental (MCT) trials for all using a customised analysis spreadsheet [43]. Pairwise comparisons were also made between observed measures relative to respective baseline values for each group. Data for BOHB and BG comparisons were log transformed for analysis to reduce bias arising from nonuniformity of error and subsequently back transformed to obtain changes in means and variations as factors. Raw data were used for comparisons between groups for Symptoms-Q and POMS-SF results. The sum of symptoms scores (Symptoms-Q) and total mood disturbance score (TMDS) of the POMS-SF were used for analysis. Total mood disturbance score (TMDS) of the POMS-SF was calculated by subtracting positive mood items from negative mood items [44]. To make inferences about true (population) values of the effect on BOHB, Symptoms-Q, and POMS of MCT relative to LCT, the uncertainty in the effect was expressed as 90% confidence limits and as likelihoods that the true value of the effect represents substantial change (harm or benefit). An effect was deemed to be *unclear* if its confidence interval overlapped the thresholds for substantiveness, that is, if the effect could be substantially positive and negative [45]. The smallest worthwhile change for between-group means for all blood and perceptual measures was calculated as 0.2 of the between-subject SD [45]. Inferences were based on threshold chances of harm of a difference between groups of 0.5% and benefit of 25%. To determine the likelihood of clinical effects, the default values and qualitative terms were set at <0.5%, *most unlikely*; 0.5 to 5%, *very unlikely*; 5% to 25%, *unlikely*; 25 to 75%, *possibly*; 75 to 95%, *likely*; 95 to 99.5%, *very likely*; and >99.5%, *most likely*. Effect sizes (ESs) were calculated using Cohens *d*, with an ES of <0.2 considered *trivial,* >0.2 *small,* >0.6 *moderate,* >1.2 *large*, and >2.0 *very large* [45].

Time to NK comparison between LCT and MCT groups was made using a Kaplan–Meier survival analysis for time to event [46]. The “event” analysed was the first recorded instance of NK (≥0.5 mmol·L^−1^ BOHB) for each participant, and a log-rank test determined the significance of the survival analysis data [46].

Finally, correlations were considered between BOHB and glucose, BOHB and symptoms (Symptom-Q), and BOHB and mood (POMS). Correlations were considered to be *trivial r* < 0.1, *small* > 0.1, *moderate* > 0.3, *large* > 0.5, *very large* > 0.7, *nearly perfect* > 0.9, or *perfect* = 1 [47]. Statistical significance of survival analyses, correlations, and additional analyses was determined by a *p* value of ≤0.05.

## 3. Results

A total of five participants withdrew during the data collection period—two from the MCT group (illness and gastrointestinal discomfort) and three from the LCT group (one due to extreme hunger, one unreported, and a third due to light-headedness and inability to concentrate). The remaining participants' (*n*=23) self-reported adherence to supplementation was 97% (combined) for both treatment groups. Thirty-two (7%) of BOHB measures failed to be recorded due to technical or operator error with the measurement device. Mean imputation analysis was used to adjust for the missing measures [48].

### 3.1. Effects on Beta-Hydroxybutyrate

**Figure 2:**
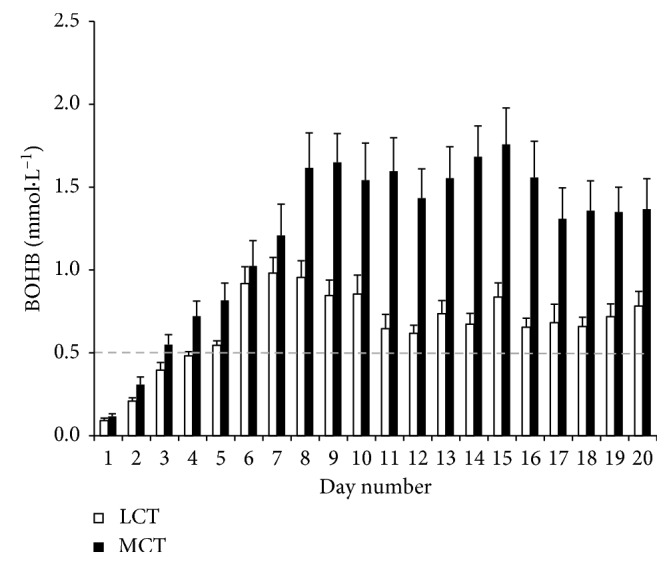
Mean BOHB for MCT versus LCT supplementation. Error bars represent SE from the mean.

Supplementing MCT resulted in consistently higher blood levels of BOHB in our cohort of healthy adults relative to LCT treatment, with higher BOHB at all time points in the MCT group (Figure 2). While clinically *trivial* effects were observed for days one to six, between-group effects for days seven to 19 were clear for MCT relative to LCT (Table 2). The magnitude of these effects was 0.2 ± 0.7 mmol·L^−1^ (days 1 to 6) and 0.8 ± 0.7 mmol·L^−1^ (days 7 to 19).

There was also a *very likely negative* effect of BOHB on glucose in both groups. That is, higher BOHB levels resulted in lower glucose levels. This was further indicated by a *very large*, significant, inverse relationship of glucose to BOHB for both MCT (*r*=−0.70,  *p*=0.0005) and LCT groups (*r*=−0.78,  *p*=0.00003).

### 3.2. Time to Nutritional Ketosis

**Figure 3:**
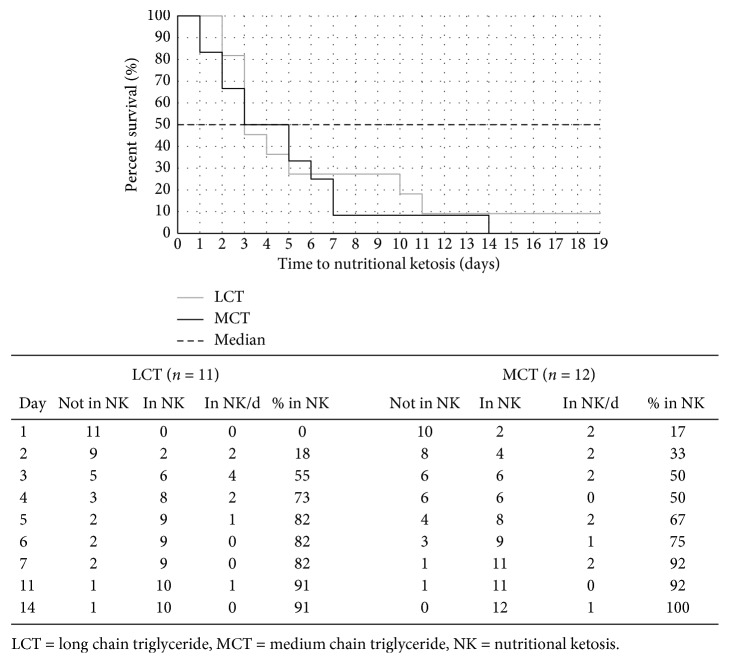
Kaplan–Meier survival graph and relative percentages of participants achieving nutritional ketosis (NK).

Overall, time to ketosis was more rapid with MCT supplementation. The achievement of NK within the first three days was higher with MCT versus LCT (17% versus 0% on day one and 33% versus 18% on day two) (Figure 3), and the mean time to NK was one day shorter with MCT supplementation but any observed differences between LCT and MCT for time to NK failed to reach significance (*p*=0.30).

### 3.3. Symptoms of Keto-Induction

**Figure 4:**
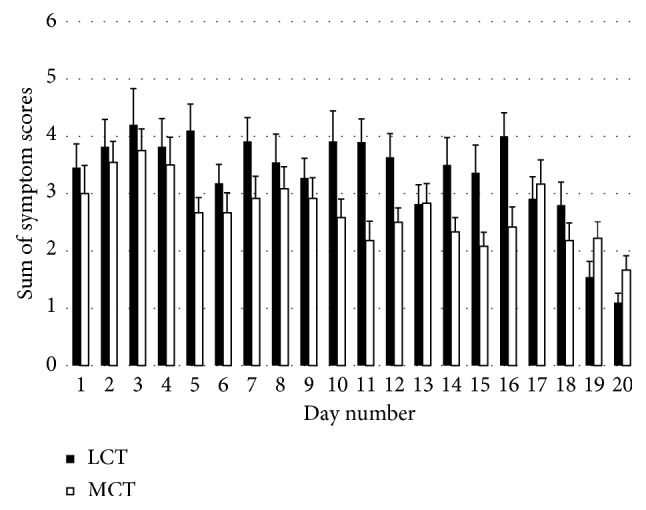
Sum of symptoms of keto-induction scores. Bars represent SE from the mean.

Supplementation with MCT versus control resulted in lower symptoms associated with keto-induction, with the mean sum of symptom scores lower in the MCT group across all time points except for days 16, 18, and 19 (Figure 4).

Effects of MCT on the change in symptoms from baseline were *possibly beneficial* for days 4, 6, 9 to 11, and 13 to 15, but on all other days, effects were *unclear* relative to LCT. Improvements in symptom scores from the preceding day indicated a *possibly beneficial* effect of MCT on 11 of 19 days. There was an *unclear* effect of BOHB as a predictor of symptoms for MCT relative to LCT (Table 2). However, there was a large inverse correlation (*r* = −0.60) observed between BOHB and Symptoms-Q in the MCT group (*p*=0.005) only. We noted a small inverse correlation between BOHB and symptoms in LCT (*r*  = −0.23), a result that failed to reach significance (*p*=0.30).

### 3.4. Mood

**Figure 5:**
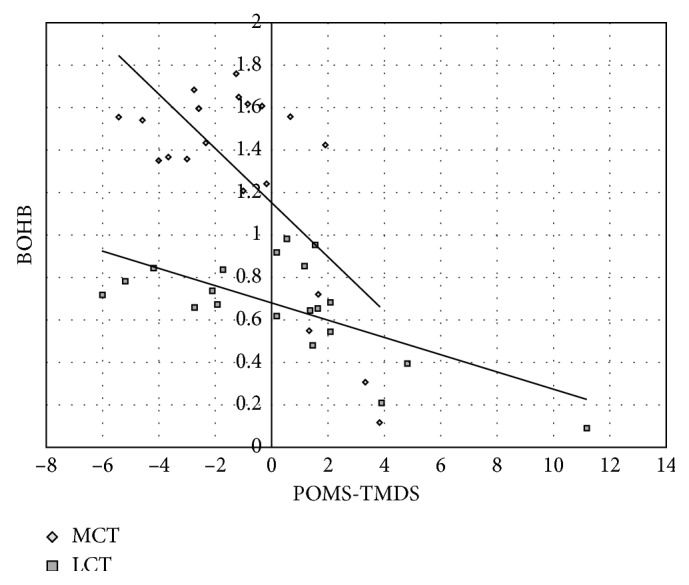
Mean BOHB compared to mean POMS-TMDS.

Mood scores were improved at all time points from baseline in both groups with generally better mood reported by the LCT group compared to the MCT. As BOHB levels increased, reported mood improved in both groups. There was a significant, *large* inverse correlation between mean BOHB and mean POMS-TMDS in both the MCT (*r*=−0.70,  *p*=0.0006) and LCT supplemented groups (*r*=−0.67,  *p*=0.001) (Figure 5). However, the effect of MCT relative to LCT on improvement in mood across all time points was *unclear* (Table 2).

When considering changes relative to the preceding day, there were approximately equal days of improvement in MCT versus LCT supplementation (nine and ten days, resp.). A *possible beneficial* effect was observed across eight days for MCT supplementation (Table 2). MCT supplementation provides *very likely beneficial* effects on TMDS when BOHB is a predictor. A *large* correlation between glucose and TMDS in both MCT (*r*=0.50,  *p*=0.02) and LCT (*r*=0.59,  *p*=0.01) was also observed. There was a *possibly beneficial* effect from MCT supplementation when glucose was used as a predictor of mood. We observed an association between mood scores and symptom scores across both groups (MCT *r*=0.61,  *p*=0.004; LCT *r*=0.63,  *p*=0.003).

## 4. Discussion

This study was the first to assess the impact of MCT on time to NK and symptoms of keto-induction and mood and, we believe, the first to specifically describe symptoms of keto-induction (keto-flu), within the first few days of a ketogenic diet.

Not surprisingly, there was a clear and significant effect of MCT supplementation on BOHB levels, relative to LCT control. MCTs are demonstrably ketogenic [29–31], and this effect was expected. All MCT participants reached NK (≥0.5 mmol·L^−1^) with one participant in the LCT group failing to reach NK. A significant effect on time to NK was not demonstrated, despite the MCT group (based on mean BOHB levels) achieving ketosis two days earlier on day 2 versus day 4, for MCT and LCT, respectively. We would consider an effect of MCT on time to NK to be likely due to the demonstrable effect on ketogenesis and ketonaemia resulting from MCT ingestion [29–31]. It must also be noted that this is likely to be a “true” effect that we observed, as the participants' BOHB levels were tested when fasted in the morning, and so, increased BOHB cannot be solely explained by transient ketonaemia resulting from MCT ingestion. This effect is further indicated by the increased magnitude of the difference in BOHB between MCT and LCT groups over the study course. Studies with larger numbers of participants will be required to test this hypothesis adequately.

Symptoms initially worsened in response to both diet interventions, but these were ameliorated by day four for MCT and day five for LCT. At this time point, mean BOHB levels were 0.8 and 0.9 mmol·L^−1^, respectively. This result might suggest that the definition for NK of ≤0.5 mmol·L^−1^ as suggested by Volek and Phinney [49] and previously used as a threshold for ketosis [15] is not sufficient as a functional measure for ketosis. We also considered that a higher entry point to NK could be 0.7 mmol·L^−1^ based on the average of the BOHB readings on the day at which symptom scores had returned to baseline and the preceding day, as the questionnaire evaluated symptoms for the previous 24 hours. With this hypothetical higher threshold for NK, achievement of NK was greater in the MCT group for the first three days, but this result was also not significant, nor was the achievement of NK appreciably different after this time. Further exploration to define NK more appropriately to functional outcomes and to determine evidence-based thresholds is warranted.

Symptoms were reduced in the MCT group relative to LCT. A *possibly beneficial* clinical effect was exhibited on symptoms by MCT application across almost all time points, and mean symptom scores returned to baseline a day earlier with MCT supplementation. While the effect of MCT when BOHB levels were used as a predictor of symptoms was *unclear,* the large inverse correlation between BOHB and symptoms in the MCT group, not observed for LCT, suggests that the increase in BOHB resulting from MCT supplementation results in improved symptoms of keto-induction. This result further suggests that there might be a threshold level of BOHB required to mitigate some of the symptoms associated with keto-induction and that the higher BOHB exhibited in the MCT group may have caused this reduction in symptoms. This is a result that warrants further exploration. Relative to the MCT group, the LCT group reported factor increases of 0.5 (*p*=0.01), 2.0 (*p*=0.01), 1.7 (*p*=0.0004), and 2.3 (*p*=0.0004) for concentration difficulties, muscle cramps, intestinal bloating, and constipation, respectively. Relative to LCT, there was a 1.7 factor increase in the incidence of abdominal pain with MCT (*p*=0.003). There are known gastrointestinal effects from ingestion of MCTs [41], and in the amounts provided to participants, it is possible that symptoms noted with a higher incidence in the MCT group, especially diarrhoea and stomach pain, resulted from the use of MCTs. Abdominal pain, halitosis, and diarrhoea were, in fact, the most commonly observed effects in the MCT group with only abdominal pain as noted, reaching significance, and halitosis (“ketone breath”) observed similarly in both groups. Reduced dosages of MCTs warrant further study to determine dose-dependent effects on symptoms and mood relative to control.

Mood scores correlated with symptom scores, but effects of MCT on mood state were *unclear*. Interestingly, mood scores were typically more positive in MCT, whereas improvement from baseline was greater with LCT compared to MCT. Day-by-day improvements in mood were similar between MCT and LCT with some benefit from MCT supplementation observed on several days (Table 2). It is unlikely that MCT would worsen mood independently, except if resultant adverse effects (such as the stomach pain noted above) was sufficient to depress mood. When BOHB was considered as a predictor variable for mood, there was a likely beneficial effect from MCT supplementation.

There were several limitations to this study. Differences in compliance may have resulted from the free-living nature of this study. We did not adjust for exercise and activity, although participants were advised to not change their current exercise habits. Standardised diets (both for male and female) were provided per age- and gender-adjusted average requirements. Thus, those participants that were more active may have exhibited differing results for BOHB, symptoms, and mood. Additionally, some participants experienced occasional difficulty taking readings with the ketometer/glucometer, resulting in several missed readings, which could have influenced results.

Because of the preliminary and exploratory nature of this study, we conducted many comparisons and hypothesis tests. We recognise the potential for multiplicity. A total of 114 pairwise comparisons were made with 28 results, or 25%, reaching clinical significance. Since it is expected by chance that 5% of tests are expected to be false positives (or 6 results), our trial shows a clear excess of positive results over the number expected if chance were the only explanation for them. While it is not possible to distinguish which differences are true positives or negatives, an excess of observed over expected suggests some of the significant differences are true. Similarly, in correlations, 3 of 5 results were significant. We also note that magnitude-based inferences are not based on *p* values, and thus, patterns of statistical significance with particular measures makes type-1 error less likely.

Furthermore, the numbers allocated to this study were limited by budgetary constraints due to its exploratory nature, and this is likely to have reduced the statistical power of our results. We conducted a retrospective power calculation. The primary outcome of interest was the incidence of nutritional ketosis. The true proportion who achieved NK at seven days was 90% in the MCT group, and 70% in the LCT group; therefore, this study would have had 25% power to detect a significant difference in these proportions (alpha level = 5%), assuming the data conforms to a chi-square distribution. It is not surprising that we did not see a statistically significant difference between the two groups in the survival analysis. The chance of a false-negative result under these circumstances would be 75%. We consider that despite the relatively small numbers in the cohort, the findings are of considerable interest to both the scientific and lay community as there has been little direct research on keto-induction and “keto-flu” specifically. We also recognise the limitations of our convenience sample arising from the snowball method of recruitment, via our networks on a first-come basis, as this led to an almost entirely female cohort.

## 5. Conclusion

MCT supplementation improves BOHB levels relative to an LCT control and has a possible, clinical application to reduce symptoms of keto-induction. It is unclear at this time whether MCTs significantly improve time to NK and mood but the large, inverse correlation between BOHB and mood disturbance scores, and the observed correlation between symptoms and mood suggest that in the context of a VLCKD, MCT supplementation may also improve mood. Due to the exploratory nature of this study, large variations between individual responses made many results unclear, especially concerning time to NK.

More research with larger sample sizes is needed in this area to elucidate the role of MCTs in a classic ketogenic diet more completely and to understand the variability between individuals in their responses to ketogenic diets.

## Figures and Tables

**Table 1:** Demographic characteristics of participants.

	MCT	LCT
Gender (M/F)	1/11	1/10
Age (years); mean (range)	40 (33 to 47)	40 (32 to 48)
Ethnicity (*n*)	European (5)	European (11)
	NZ Maori (2)	
	Pacific Island (3)	
	Chinese (1)	
	Other Asian (1)	

MCT: medium chain triglyceride; LCT: long-chain triglyceride; M: male; F: female.

**Table 2:** Standardised effects for mean differences (LCT − MCT) in mood state, BOHB, and blood glucose for days 1 to 19.

	Day →	Baseline	1	2	3	4	5	6	7	8	9	10	11	12	13	14	15	16	17	18	19
∆ symptoms from baseline	Standard difference in means		−0.08	0.19	−0.27	−0.02	−0.17	0.00	0.03	−0.28	−0.24	−0.22	0.15	−0.12	−0.26	−0.36	0.23	0.09	0.79	0.67	0.86
CL 90%	±		0.86	0.99	0.66	0.70	0.67	0.83	0.77	0.74	0.72	0.66	0.70	0.76	0.75	0.74	0.89	0.83	0.65	0.70	0.66
Clinical inference			=			+	−	+	=		+			=	+			=			

∆ symptoms by day	Standard difference means		0.06	−0.16	−0.03	−0.24	0.34	−0.24	0.38	−0.10	−0.26	−0.37	0.36	0.31	−0.18	−0.19	−0.15	0.60	−0.26	0.30	0.06
CL 90%	±		0.85	0.67	0.49	0.67	0.54	0.49	0.50	0.56	0.53	0.73	0.62	0.64	0.50	0.43	0.64	0.60	0.68	0.48	0.41
Clinical inference			=	+			=	+	=	+			=	=	+			=	+	=	

∆ POMS from baseline	Standard difference in means		0.46	0.26	0.65	0.35	0.62	0.40	0.34	0.71	0.11	0.23	0.33	0.28	0.45	0.54	0.44	0.34	0.48	0.64	0.61
CL 90%	±		0.71	0.77	0.80	0.77	0.61	0.80	0.90	0.84	0.84	0.83	0.84	0.79	0.86	0.80	0.73	0.60	0.86	0.80	0.21
Clinical Inference			=																		

∆ POMS by day			0.46	−0.20	0.25	−0.17	0.27	−0.22	−0.06	0.37	−0.60	0.12	0.10	−0.06	0.17	0.09	−0.10	−0.10	0.15	0.16	−0.03
CL 90%	±		0.71	0.35	0.37	0.41	0.59	0.62	0.38	0.57	0.30	0.47	0.35	0.43	0.33	0.27	0.58	0.35	0.63	0.27	0.23
Clinical Inference			=	+	=	+	=	+		=	++	=	=	+	=	−−−	+		=		−−

BOHB	Difference in means as a factor	1.16	1.24	1.16	1.06	1.00	1.04	1.48	1.90	1.87	2.45	1.91	1.85	2.31	1.84	1.76	2.06	1.67	1.93	1.71	1.16
CL 90%	×∕÷	1.75	1.71	1.61	1.68	1.74	1.79	1.89	1.79	1.90	1.91	1.71	1.76	1.69	1.82	1.86	2.01	1.98	1.90	1.97	1.75
Nonclinical inference		=							++		+++	++		+++	++						=

Glucose	Difference in means as a factor	0.94	0.99	0.99	0.98	1.00	0.98	0.96	0.95	1.03	0.93	0.88	1.04	0.85	1.00	0.98	0.94	0.94	0.93	1.01	0.94
CL 90%	×∕÷	1.11	1.14	1.09	1.10	1.08	1.10	1.11	1.10	1.11	1.09	1.11	1.09	1.26	1.10	1.11	1.07	1.07	1.07	1.08	1.11
Nonclinical inference		+	=							++	−−		+++	=					−	++	+

Standardised effects for mean changes (LCT − MCT) in Symptom-Q and POMS-TMDS for days 1 to 19 relative to baseline measures and for day-to-day changes. Clinical inferences based on threshold chances of harm and benefit of 25% and 0.5%. Nonclinical inferences based on threshold chances of 5% for substantial magnitudes. Positive or clinically beneficial: +++, very likely; ++, likely; +, possibly; =, unclear; negative or not clinically beneficial: −, possibly; −−, likely; −−−, very likely.

## Data Availability

Data are available upon request.
